# Correlation Between Quantitative Uptake of ^99m^TC-DPD and Echocardiographic Parameters in Cardiac ATTR: A Novel Follow-Up Strategy

**DOI:** 10.3389/fcvm.2021.663929

**Published:** 2021-10-15

**Authors:** Mehmet Harapoz, Scott Evans, Paul Geenty, Fiona Kwok, Graeme Stewart, Mark S. Taylor, David Farlow, Liza Thomas

**Affiliations:** ^1^Department of Cardiology, Westmead Hospital, Sydney, NSW, Australia; ^2^Westmead Clinical School, University of Sydney, Sydney, NSW, Australia; ^3^Department of Nuclear Medicine, Westmead Hospital, Sydney, NSW, Australia; ^4^Westmead Amyloidosis Service, Westmead Hospital, Sydney, NSW, Australia; ^5^Department of Haematology, Westmead Hospital, Sydney, NSW, Australia; ^6^Department of Clinical Immunology and Allergy, Westmead Hospital, Sydney, NSW, Australia; ^7^South Western Sydney Clinical School, University of New South Wales, Sydney, NSW, Australia

**Keywords:** nuclear cardiology and PET, ^99m^Tc-DPD scintigraphy, echocardiography, amyloidosis, cardiomyopathy

## Abstract

**Aims:** There has been a paradigm shift in diagnosis of cardiac transthyretin amyloidosis (ATTR) with non-invasive techniques including technetium-99m 3,3-diphosphono-1,2-propanodicarboxylic acid (^99m^Tc-DPD) bone scintigraphy. We evaluated structural and functional biventricular alterations by transthoracic echocardiography (TTE) and determined the correlation with ^99m^Tc-DPD tracer uptake in ATTR.

**Materials and Methods:** ATTR patients (wild-type, hereditary or asymptomatic transthyretin [*TTR*] variant carriers) with ^99m^Tc-DPD and TTE were selected; ^99m^Tc-DPD uptake was analyzed quantitatively. TTE assessment of left ventricle (LV) and right ventricle (RV) parameters was performed.

**Results:** Forty ATTR patients (wild-type *n* = 17; hereditary ATTR and *TTR* variant carriers *n* = 23; median age 68.8 ± 22 years) were included. TTE parameters displaying good correlation with ^99m^Tc-DPD tracer uptake included LV average wall thickness (*r* = 0.837), LV indexed mass (LVMI; *r* = 0.802), RV wall thickness (*r* = 0.610), average e' (*r* = −0.830), E/e' ratio (*r* = 0.786), LV global longitudinal strain (GLS; *r* = 0.714) and RV GLS (*r* = 0.632; *p* < 0.001 for all). Hereditary ATTR and *TTR* variant carriers without cardiac tracer uptake had normal echocardiographic parameters. Receiver operating characteristic curves demonstrated strong diagnostic accuracies for structural (LV wall thickness, LVMI and RV wall thickness; area under the curve (AUC) of 0.96 for all) and functional (LV and RV GLS; AUC of 0.86 and 0.88, respectively) parameters.

**Conclusion:** Good correlations between TTE biventricular structural and functional parameters were demonstrated with quantitative ^99m^Tc-DPD uptake. Echocardiography may potentially assume a significant role in longitudinal follow-up for monitoring disease progression and for evaluating treatment response.

## Introduction

Systemic amyloidosis is a multiorgan disease resulting from extracellular deposition of insoluble amyloid fibrils, formed from aggregated, misfolded proteins (1, 2). Cardiac amyloidosis is predominantly caused by light chain amyloid (AL) and transthyretin amyloidosis [ATTR] (2); other amyloid subtypes are less prevalent and rarely demonstrate cardiac involvement (2).

ATTR subtypes include wild-type and hereditary, both displaying heterogeneity in clinical symptoms and organ involvement (1, 2). Cardiac involvement in ATTR is common, with median survival without treatment of 2–4 years, presenting commonly with heart failure with preserved ejection fraction, conduction disorders and atrial fibrillation (1, 3–9).

Cardiac amyloidosis diagnosis can be difficult, with definitive diagnosis usually based on tissue immunohistochemistry and/or mass spectrometry proteomic analysis of cardiac biopsy specimens (10, 11). Cardiac amyloidosis subtype identification is crucial, particularly as novel treatment options emerge for ATTR (12, 13). Several non-invasive screening tests are now available including transthoracic echocardiography (TTE) and cardiac magnetic resonance imaging (12). Recently, technetium-99m 3,3-diphosphono-1,2-propanodicarboxylic acid (^99m^Tc-DPD) scintigraphy emerged as an important diagnostic modality with strong tracer avidity for the transthyretin protein, with high sensitivity and specificity (10, 11, 14, 15). Most cardiac amyloidosis studies using ^99m^Tc-DPD scintigraphy employed qualitative visual grading or semi-quantitative grading with heart-to-body ratios (15, 16). Few studies employed quantitative ^99m^Tc-DPD tracer uptake analysis, and consequently, little is known about tracer uptake correlation with specific structural and functional cardiac changes (16, 17).

This study evaluated echocardiographic biventricular structural and functional involvement and determined their correlation with ^99m^Tc-DPD tracer uptake quantitative analysis. We hypothesized that the extent of structural and functional abnormalities on TTE should be commensurate with ^99m^Tc-DPD tracer uptake amount.

## Materials and Methods

We retrospectively reviewed 42 consecutive patients with wild-type, hereditary ATTR and asymptomatic transthyretin (*TTR*) variant carriers who attended the Westmead Hospital Amyloidosis Clinic, a statewide referral service for amyloidosis patient evaluation in New South Wales, Australia. Ethics was submitted and approved by the Western Sydney Local Health District (WSLHD) Research Ethics and Governance Committee in keeping with the Declaration of Helsinki. Two patients were excluded as ^99m^Tc-DPD reference imaging was not available for quantitative analysis.

**Figure 1:**
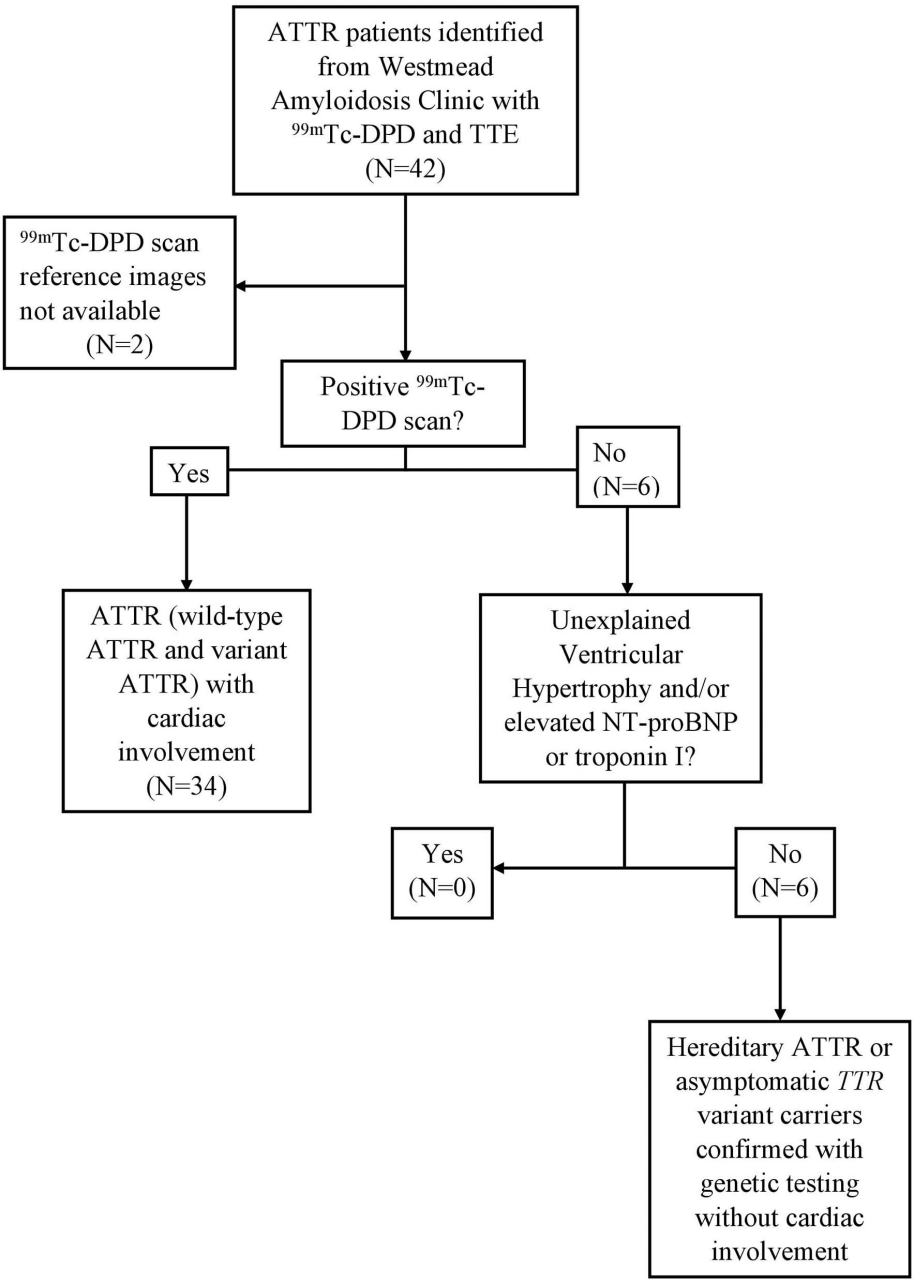
Study protocol, eligibility and cardiac involvement assessment. ATTR patients with ^99m^Tc-DPD scintigraphy and TTE within a 12-month window. In total, 40 ATTR patients were included, 34 with ^99m^Tc-DPD scintigraphy evidence of cardiac involvement. ATTR, Transthyretin Amyloidosis; ^99m^Tc-DPD, technetium-99m 3,3-diphosphono-1,2-propanodicarboxylic acid; TTE, Transthoracic echocardiogram; NT-pro-BNP, N-terminal-pro hormone brain natriuretic peptide.

In total, 40 patients (wild-type *n* = 17; hereditary ATTR and asymptomatic *TTR* variant carriers *n* = 23) were included for analysis after screening to ensure absence of a monoclonal gammopathy. All had undergone TTE and ^99m^Tc-DPD scintigraphy within a 12-month period. Figure 1 demonstrates the algorithm to identify cardiac involvement. Thirty-four patients were determined to have cardiac ATTR on ^99m^Tc-DPD scintigraphy; the remaining six patients had hereditary ATTR or were asymptomatic *TTR* variant carriers confirmed by ATTR genotyping, without cardiac involvement. Both *TTR* variant carriers and hereditary ATTR patients had positive family histories and were identified to have an amyloidogenic mutation, with hereditary ATTR patients additionally having clinical symptoms or proven amyloid deposition (Val122Ile *n* = 5, Val71Ala *n* = 1, Ala97Ser *n* = 3, Val30Met *n* = 6, Ser52Pro *n* = 1, Thr60Ala *n* = 5, Ser77Tyr *n* = 2). One patient identified to have cardiac involvement had mild aortic stenosis. All patients had serum creatinine (allowing derivation of estimated glomerular filtration rate), troponin I, N-terminal-pro hormone brain natriuretic peptide (NT-proBNP) and electrocardiography. Demographic and clinical information were collected for all patients. Sixteen patients had mild hypertension (14/34 in those with cardiac amyloidosis and 2/6 in those without).

### Electrocardiogram

Electrocardiograms were evaluated for rhythm, PR interval, QRS duration, low voltage and pseudoinfarction pattern. Low voltage pattern was defined as either voltage <1 millivolt in all precordial leads or <0.5 millivolt in all limb leads (18). Pseudoinfarction pattern was defined as pathologic Q or QS waves in 2 consecutive leads in the absence of left bundle branch block or ischemic heart disease (18).

### Echocardiography

A comprehensive TTE was performed using commercially available ultrasound systems (General Electric Vivid E9/E95; Horton, Norway); examinations included 2-dimensional, color and Doppler echocardiography, performed in accordance with the American Society of Echocardiography and European Association of Cardiovascular Imaging recommendations (19). Dedicated left ventricle (LV) and right ventricle (RV) views were obtained at high frame rates (>55 frames per second) by experienced sonographers with optimisation of ventricular images. All images were stored as raw digital data for offline analysis and the investigators performing the measurements were blinded to the scintigraphy results. All investigators received extensive training at a tertiary center experienced in echocardiography by senior clinicians.

LV interventricular septum and posterior wall thickness were measured from the parasternal long axis view in end-diastole (19). Average wall thickness was calculated as (LV interventricular septum thickness + LV posterior wall thickness)/2. LV end-diastolic and end-systolic volumes were measured using the modified method of disks, from apical 4 and 2 chamber views, allowing LV ejection fraction (LVEF) calculation (19).

LV mass was derived using the Devereux formula and indexed to body surface area, obtaining LV indexed mass (LVMI) (20). LV wall thickness ≥12 mm was considered increased and indicative of cardiac amyloidosis (21). The upper limit of normal LVMI was defined as 115 g/m^2^ for males and 95 g/m^2^ for females (20).

For LV diastolic function, peak E and A wave velocities were obtained using pulsed wave Doppler, with the sample volume placed at the mitral leaflet tips (22). Tissue Doppler velocities were obtained from the septal and lateral mitral annulus and e' velocities were obtained (22). Septal and lateral e' velocities were averaged and utilized to calculate E/e' ratio, as a surrogate marker for elevated LV filling pressure (22).

The RV focused view was used to obtain end-diastolic and end-systolic areas to calculate fractional area change (23). Tricuspid annular plane systolic excursion was measured using an M-mode cursor through the lateral tricuspid annulus (23). RV s' velocity was obtained using tissue Doppler from the lateral tricuspid annulus (23). RV wall thickness was measured from the subcostal view, in end diastole (23).

Biventricular 2-dimensional speckle tracking strain analysis was performed using specialized computer software (EchoPac Version 203; GE systems).

LV strain analysis was performed from the 3 apical views (apical 4, 2 and long axis) by tracing the endocardium (20). A region of interest was created by the software during offline analysis and adjusted to accommodate myocardial thickness, providing an 18-segment LV model (6 segments in each apical view). Manual adjustment was performed in segments failing to track and allowed up to 2 segments to be excluded from any apical view. An average of 3 measurements was performed for patients in sinus rhythm and an average of 5 measurements for those in atrial fibrillation. LV global longitudinal strain (GLS) was calculated as an average of the 18 segments.

RV strain analysis was performed by tracing the endocardium in the RV focused view (20). A region of interest was defined by the software and adjusted for myocardial thickness. RV strain was calculated from a 6-segment model as RV GLS (3 segments each from the free wall and septum) (20). Additionally, RV free wall longitudinal strain (FWS) was calculated as the average of the 3 free wall segments.

### Analysis of ^99m^Tc-DPD Scintigraphy

^99m^Tc-DPD scintigraphy was performed using Siemens Symbia single-photon emission computed tomography-computerized tomography (SPECT-CT) machine (Erlangen, Germany) with injection of 900MBq ^99m^TC-DPD, which was followed 1 h later by SPECT of the thorax and upper abdomen including heart, lungs and liver in the field of view. A SPECT-CT of this region and whole-body sweep were acquired 3 h after injection. The SPECT-CT examination was utilized for quantification with attenuation correction, scatter correction and resolution recovery options applied to the data set. Reference standard for uptake was the aortic arch blood pool (24). Cardiac ^99m^Tc-DPD uptake was quantified for defined volumetric regions of the cardiac chambers, including the myocardium, as values of counts per pixels as the ratio of signal from the myocardial region of interest to the reference blood pool signal and is similar to that previously described using ^99m^Technetium-hydroxymethylene diphosphonate scintigraphy (24, 25). The ratio compared tracer uptake in individual cardiac chambers to the level of circulating radioactivity in the circulating blood pool (25). In this study, a positive result was defined as a ratio in the heart greater than 1.1 or 110% of the tracer uptake of blood pool activity (24). This approach used custom software developed by the Department of Nuclear Medicine at Westmead Hospital, New South Wales, Australia, in Interactive Data Language providing quantitative values for tracer uptake. Each ventricle was quantified individually for comparison with the relevant echocardiographic parameters.

### Intra- and Interobserver Variability

Intra- and interobserver variability were evaluated for LV wall thickness, LVMI and strain analysis. Intraobserver variability was evaluated on 20 randomly selected patients by the same observer at least 2 weeks after the initial measurements were performed. Interobserver variability was performed by two trained investigators on the same 20 patients. Intraobserver duplicate analysis was performed in 24 patients for ^99m^TC-DPD scintigraphy.

### Statistical Analysis

Normality of data was tested using the Shapiro–Wilk test. Continuous data has been expressed as either the mean and standard deviation or as a median with interquartile range. Categorical variables have been expressed as percentages. TTE parameter measurements were compared to quantitative ^99m^Tc-DPD scintigraphy measurements using non-parametric Spearman ranked correlation and Fisher's exact test. Scatterplots were created to illustrate correlations. The Mann–Whitney U test was used to compare patients with and without ^99m^Tc-DPD scintigraphy evidence of cardiac involvement. Receiver operating characteristic curves were used to assess parameter sensitivity and specificity, then further analyzed with DeLong's method for significant differences (26). Bland–Altman analysis was used to evaluate intra- and interobserver variability. A *p*-value < 0.05 was considered statistically significant. All statistical analysis was performed using SPSS software version 23.0 (SPSS, Inc., Chicago, Illinois) and MedCalc software version 14.8 (MedCalc, Belgium).

## Results

Forty patients comprised the study cohort (30 males; median age 68.8 ± 22 years) with 34 patients with cardiac involvement. The remaining 6 patients were genotyped as carrying an amyloidogenic *TTR* variant with one having peripheral neuropathy. TTE and ^99m^Tc-DPD scintigraphy were performed with a median time of 7 days between scans (inter-quartile range 99.8 days); 88% of patients had scans within 4 months of each other. Six patients with pacemakers were excluded from the electrocardiogram analysis. Demographic, biochemical, electrocardiogram and TTE data for the group are presented in Tables 1A,B. Fourteen of the 34 patients with cardiac amyloidosis had mild hypertension and 6 had atrial flutter/fibrillation.

**Table 1:** **(A)** Demographics, biochemical, electrocardiogram and **(B)** echocardiographic parameters for ATTR patients.

**Parameter**	**ATTR patients (*n* = 40)**
**A**	
Male	30 (75%)
Age (years)	68.8 [22]
Height (cm)	172 [16.1]
Weight (kg)	75.5 [27.9]
Body mass index (kg/m^2^)	26.3 [4.47]
Body surface area (m^2^)	1.87 [0.43]
Systolic blood pressure (mmHg)	124 [17]
Diastolic blood pressure (mmHg)	77 [10.5]
Duration between scans (days)	7 [99.8]
eGFR (mL/min/1.73m^2^)	83 [28.5]
NT-proBNP (ng/L)	1,023.5 [3407]
Troponin I (ng/L)	38 [70]
Heart rate (bpm)	70 [17.8]
Sinus rhythm	28 (70%)
Paced rhythm	6 (15%)
Atrial fibrillation/flutter	6 (15%)
PR interval (ms)	180 [80]
QRS duration (ms)	80 [40]
Low-voltage pattern	2 (5%)
Pseudo infarction pattern	11 (27.5%)
**B**	
LV average wall thickness (mm)	15 [7]
LVMI (g/m^2^)	139.8 [94.66]
Peak E velocity (cm/s)	74 [29]
Peak A velocity (cm/s)	44 [37]
E/A	1.6 [1.8]
Average e' (cm/s)	6 [3.9]
E/average e'	12.8 [12.4]
LVEF (%)	55 [11.75]
LV GLS (%)	−13.9 [8.6]
RV wall thickness (mm)	8 [5]
Fractional area change (%)	37.6 [17.8]
TAPSE (mm)	19.5 [6]
RV s' velocity (cm/s)	10 [3.5]
RV FWS (%)	−20.2 [9]
RV GLS (%)	−16.3 [8.6]

*Values are expressed as number (percentage) or median [interquartile range] for Tables 1A,B. ^†^ATTR, transthyretin amyloidosis; eGFR, estimated glomerular filtration rate; NT-pro-BNP, N-terminal-pro hormone brain natriuretic peptide; LVEF, left ventricular ejection fraction; LV, left ventricle; GLS, global longitudinal strain; LVMI, left ventricular indexed mass; RV, right ventricle; TAPSE, Tricuspid annular plane systolic excursion; FWS, free wall strain.

Echocardiographic LV parameters were obtained for all patients. Due to limited image quality, RV fractional area change, RV FWS and RV GLS could not be obtained in two patients and RV wall thickness and RV s' velocity could not be performed in one patient.

The ^99m^Tc-DPD scintigraphy signal obtained for each ventricle demonstrated a median LV value of 272% (interquartile range 462%) and median RV value of 203% (interquartile range 265%). There was a significant correlation of NT-proBNP levels with LV and RV ^99m^Tc-DPD tracer uptake (Table 2).

**Table 2:** Correlation of clinical and echocardiographic parameters with ^99m^Tc-DPD tracer uptake in ATTR patients (*n* = 40).

**Parameter**	**Left ventricular scintigraphy uptake**		**Right ventricular scintigraphy uptake**	
	***r***	***p*-value**	***r***	***p*-value**
NT-proBNP (ng/L)	0.812	<0.001	0.756	<0.001
Troponin I (ng/L)	0.677	<0.001	0.608	<0.001
LV average wall thickness (mm)	0.837	<0.001	–	–
LVMI (g/m^2^)	0.802	<0.001	–	–
Peak E velocity (cm/s)	0.404	0.011	–	–
Average e' (cm/s)	−0.830	<0.001	–	–
E/average e'	0.786	<0.001	–	–
LVEF (%)	−0.548	<0.001	–	–
LV GLS (%)	0.714	<0.001	–	–
RV wall thickness (mm)	–	–	0.610	<0.001
Fractional area change (%)	–	–	−0.241	0.146
TAPSE (mm)	–	–	−0.398	0.011
RV s' velocity (cm/s)	–	–	−0.406	0.010
RV FWS (%)	–	–	0.463	0.003
RV GLS (%)	–	–	0.632	<0.001

*Significant if p < 0.05. ^99m^Tc-DPD, technetium-99m 3,3-diphosphono-1,2-propanodicarboxylic acid scintigraphy; ATTR, transthyretin amyloidosis; NT-pro-BNP, N-terminal-pro hormone brain natriuretic peptide; LV, left ventricle; LVMI, left ventricular indexed mass; LVEF, left ventricular ejection fraction; GLS, global longitudinal strain; RV, right ventricle; FWS, free wall strain.

**Figure 2:**
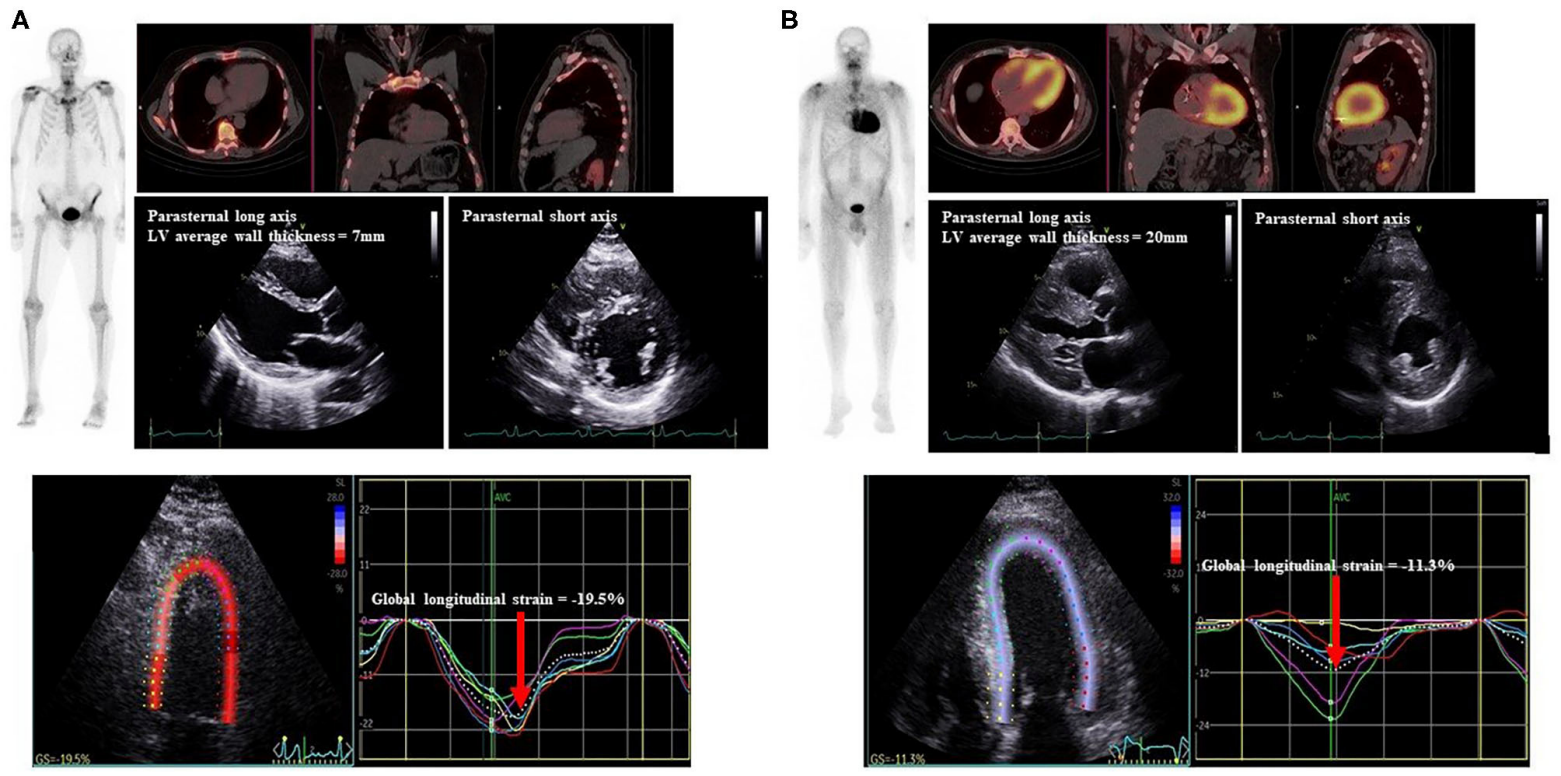
Representative Echocardiogram and ^99m^Tc-DPD scintigraphy of study patients. Echocardiograms with parasternal long and short axis views with LV wall thickness measured, demonstration of LV GLS (red arrow), ^99m^Tc-DPD whole body sweep and SPECT-CT demonstrating **(A)** negative scintigraphy, normal LV wall thickness and GLS and **(B)** positive scintigraphy, increased LV wall thickness and reduced LV GLS. ^99m^Tc-DPD, Technetium-99m 3,3-diphosphono-1,2-propanodicarboxylic acid; LV, Left ventricle; GLS, global longitudinal strain; SPECT-CT, single-photon emission computed tomography-computerized tomography.

We examined the relationship between structural and functional echocardiographic parameters for LV, RV and regional myocardial ^99m^Tc-DPD uptake (Figure 2, Table 2).

**Figure 3:**
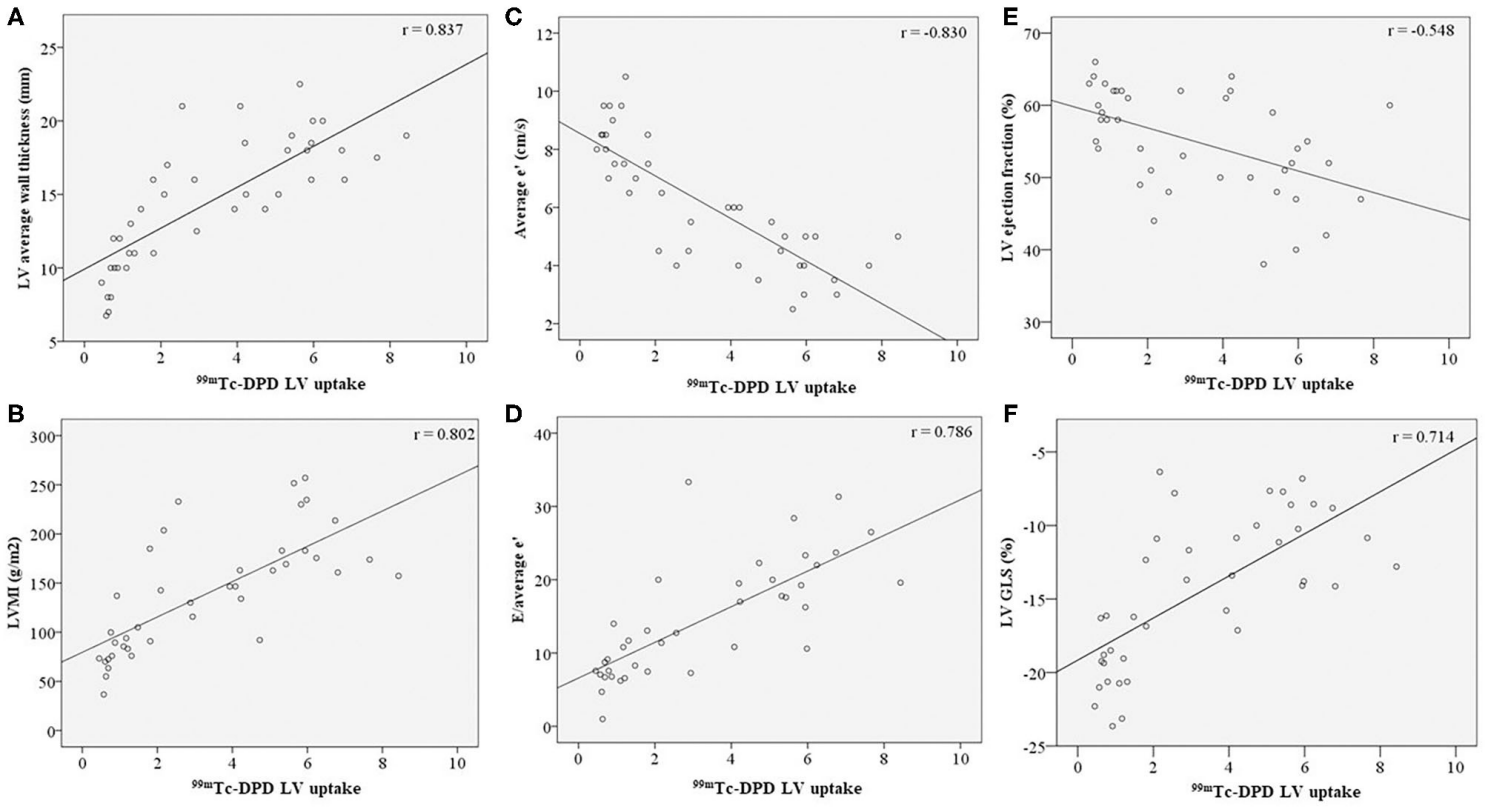
Correlation of LV structural and functional parameters with ^99m^Tc-DPD uptake. ^99m^Tc-DPD LV uptake correlation with **(A)** LV average wall thickness, **(B)** LVMI, **(C)** Average e', **(D)** E/average e', **(E)** LV ejection fraction and **(F)** LV GLS. Line of best fit plotted on all graphs. ^99m^Tc-DPD, Technetium-99m 3,3-diphosphono-1,2-propanodicarboxylic acid; LV, Left ventricle; LVMI, left ventricular indexed mass; GLS, global longitudinal strain.

Echocardiographic LV structural parameters of average wall thickness and LVMI demonstrated significant correlation with ^99m^Tc-DPD tracer uptake (Figures 3A,B). LV diastolic functional parameters of average e'and E/e' demonstrated good correlation with ^99m^Tc-DPD uptake (Figures 3C,D); however, peak E velocity demonstrated weak correlation. Of the LV systolic functional parameters evaluated, LVEF demonstrated a weak inverse correlation while LV GLS demonstrated a strong correlation (Figures 3E,F).

**Figure 4:**
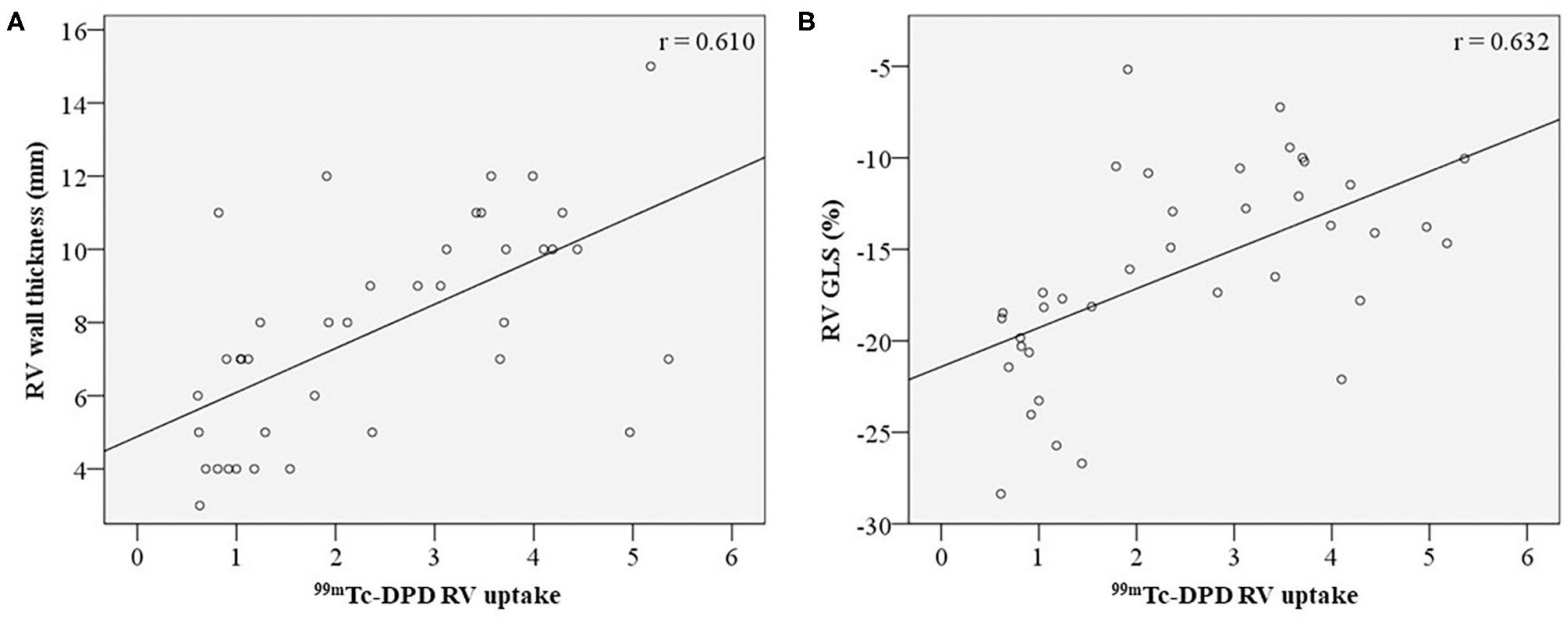
Correlation of RV structural and functional parameters with ^99m^Tc-DPD uptake. ^99m^Tc-DPD RV uptake correlation with RV wall thickness **(A)** and RV GLS **(B)**. ^99m^Tc-DPD, Technetium-99m 3,3-diphosphono-1,2-propanodicarboxylic acid; RV, Right ventricle; GLS, global longitudinal strain.

RV wall thickness demonstrated positive correlation with RV ^99m^Tc-DPD uptake (Figure 4A). Of the RV functional parameters evaluated (fractional area change, tricuspid annular plane systolic excursion, RV s' velocity, RV FWS and RV GLS), RV GLS was the only parameter demonstrating a modest positive correlation (*r* = 0.632, *p*-value < 0.001) (Figure 4B), while other RV functional parameters demonstrated weak correlations (Table 2).

Of the 6 patients without ^99m^Tc-DPD scintigraphic evidence of cardiac involvement, all demonstrated normal biventricular wall thickness and function (Table 3). Patients with ^99m^Tc-DPD scintigraphic evidence of cardiac ATTR had reduced LV GLS, increased average wall thickness and LVMI compared to those without. Patients with ^99m^Tc-DPD scintigraphic evidence of cardiac ATTR were found similarly to have RV wall thickness increased and functional RV parameters reduced (Table 3).

**Table 3:** Comparison of echocardiographic parameters between ATTR patients with and without cardiac involvement.

**Parameter**	**Patients with cardiac involvement** **(*n* = 34)**	**Patients without cardiac involvement** **(*n* = 6)**	***p*-value**
LV average wall thickness (mm)	15.5 (3.7)	8.6 (1.6)	<0.001^*^
LVMI (g/m^2^)	151.7 (54.4)	65.5 (18.3)	<0.001^*^
Peak E velocity (cm/s)	80 (23)	65 (7)	0.098
Average e' (cm/s)	6 (2)	9 (1)	0.001^*^
E/average e'	16.1 (7.6)	6.3 (2.7)	0.003^*^
LVEF (%)	54.2 (7.4)	59.2 (4)	0.099
LV GLS (%)	−13.5 (4.9)	−19.6 (1)	0.006^*^
RV wall thickness (mm)	8.5 (2.6)	4 (0.6)	<0.001^*^
Fractional area change (%)	35.1 (12.4)	46.6 (7.8)	0.020^*^
TAPSE (mm)	18.6 (5.2)	21.5 (1.9)	0.095
RV s' velocity (cm/s)	9.3 (3.1)	11.3 (1.5)	0.062
RV FWS (%)	−19.4 (6.8)	−25.2 (4.2)	0.025^*^
RV GLS (%)	−15.2 (5.5)	−21 (2.3)	0.006^*^

* *Significant if p < 0.05. ^†^Values are expressed as mean (standard deviation). ^‡^ATTR, transthyretin amyloidosis; LV, left ventricle; LVMI, left ventricular indexed mass; LVEF, left ventricular ejection fraction; GLS, global longitudinal strain; RV, right ventricle; TAPSE, tricuspid annular plane systolic excursion; FWS, free wall strain*.

**Figure 5:**
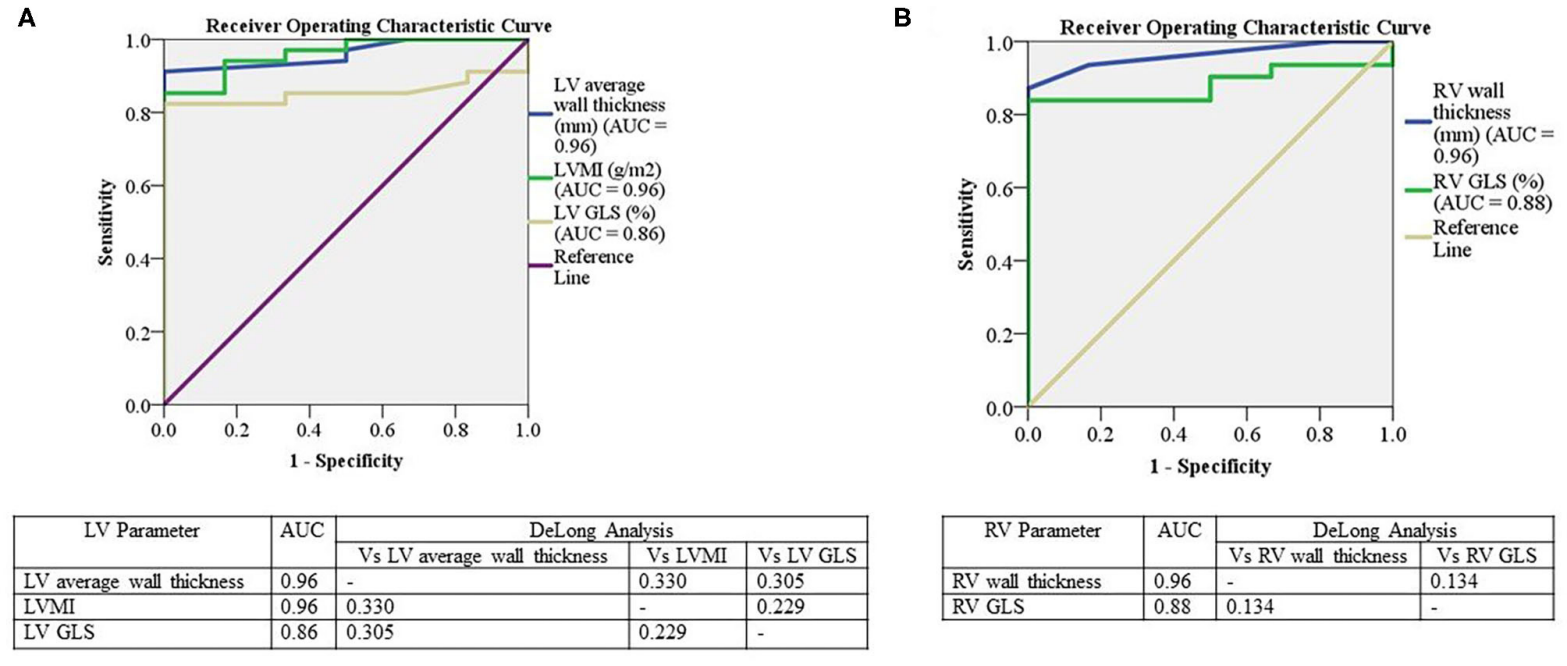
Receiver operator curves for the relationship between LV and RV parameters to ^99m^Tc-DPD uptake. Receiver operator curves for the relationship between LV ^99m^Tc-DPD uptake with LV average wall thickness, LVMI and LV GLS **(A)** and RV ^99m^Tc-DPD uptake with RV wall thickness and RV GLS **(B)**. Significant if *p* < 0.05. LV, Left ventricle; AUC, area under the curve; LVMI, left ventricular indexed mass; GLS, global longitudinal strain; RV, Right ventricle; Vs, Versus; ^99m^Tc-DPD, Technetium-99m 3,3-diphosphono-1,2-propanodicarboxylic acid.

Receiver operating characteristic analysis was performed on echocardiographic LV and RV parameters to determine diagnostic accuracy in identifying cardiac ATTR based on ^99m^Tc-DPD scintigraphy. Average LV wall thickness and LVMI demonstrated the highest diagnostic accuracy (area under the curve = 0.96 for both); there was moderate sensitivity (77%) and high specificity (100%) for a cutoff of 12.2 mm for wall thickness, whereas LVMI had lower sensitivity (71%), but similar specificity (100%) for a cutoff value of 110 g/m^2^ (Figure 5A). LV GLS had an area under the curve of 0.86, with moderate sensitivity (79%) and high specificity (100%) using a cutoff value of GLS of −17% (Figure 5A).

On assessment of RV parameters, RV wall thickness demonstrated an area under the curve of 0.96 with high sensitivity (87.1%) and specificity (100%) using a cutoff value of 5.5 mm (Figure 5B). RV GLS had an area under the curve of 0.88 with sensitivity and specificity of 83% using a cutoff value of −18.6% (Figure 5B). There was no significant difference in LV or RV parameters using DeLong's method [Figures 5A,B] (26).

### Reproducibility

Intra- and interobserver variability for LV average wall thickness, LVMI and biventricular strain analysis demonstrated overall low variability (Table 4). Intraobserver duplicate ^99m^TC-DPD scintigraphy analysis in all regions demonstrated a coefficient of correlation >0.93 (24).

**Table 4:** Bland–Altman analysis for intra- and interobserver variability.

	**Echocardiographic parameters**	**MD**	**Limits of agreement (MD ±1.96 SD)**
Intraobserver	LV average wall thickness (mm)	0.005	0.124 to −0.114
	LVMI (g/m^2^)	0.435	12.777 to −11.907
	LV GLS (%)	0.097	1.237 to −1.043
	RV FWS (%)	0.164	2.800 to −2.472
	RV GLS (%)	−0.138	1.257 to −1.533
Interobserver	LV average wall thickness (mm)	0.02	0.167 to −0.127
	LVMI (g/m^2^)	−0.997	7.853 to −9.847
	LV GLS (%)	0.18	1.906 to −1.546
	RV FWS (%)	0.002	3.774 to −3.770
	RV GLS (%)	−0.79	1.601 to −3.181

*Significant if p < 0.05. p-value was not significant for all above measurements. MD, mean difference; SD, standard deviation; LV, left ventricle; LVMI, left ventricular indexed mass; GLS, global longitudinal strain; RV, right ventricle; FWS, free wall strain.

## Discussion

The salient findings from this study are the demonstration of robust correlation of LV and RV structural and functional echocardiographic parameters with ^99m^Tc-DPD tracer uptake analysis, using ^99m^Tc-DPD SPECT-CT as a quantitative measure of ATTR deposition in myocardial tissue. Our results demonstrate that increasing ^99m^Tc-DPD tracer uptake is associated with greater structural change with increased biventricular wall thickness. Decreased LV diastolic and systolic function, as well as RV systolic function, were also associated with ^99m^Tc-DPD tracer uptake. In addition, we observed hereditary ATTR patients and asymptomatic *TTR* variant carriers without myocardial ^99m^Tc-DPD uptake demonstrating normal echocardiographic structure and function.

Structural echocardiographic parameters (LV and RV wall thickness and LVMI) demonstrated stronger correlation with ^99m^Tc-DPD uptake compared to functional echocardiographic parameters. This stronger correlation is probably because ^99m^Tc-DPD uptake reflects the amount of myocardial amyloid content and not the functional consequences thereof. Moreover, coexistent cardiac pathologies not reflected by ^99m^Tc-DPD tracer uptake could impact on functional myocardial properties.The presence of hypertension can result in increased LV wall thickness (27). Our group has previously demonstrated a reduction in LV GLS in hypertensive patients as well as those with AL amyloidosis vs. controls (28). Sixteen patients (14/34 in the cardiac amyloidosis group and 2/6 without cardiac involvement) had hypertension. The coexistent hypertension was only mild (mean systolic BP was 126 mmHg), but could have altered LV GLS. Such a confounding effect may only reduce correlations with tracer uptake on ^99m^Tc-DPD scintigraphy. Despite this, correlations demonstrated were robust between LV wall thickness and GLS and LV tracer uptake on ^99m^Tc-DPD scintigraphy.

It has previously been demonstrated that patients with atrial fibrillation may have impaired LV GLS compared to those in sinus rhythm independent of sex, age, heart rate, LVEF and LV mass (29). A variation in LV GLS should reduce correlation with tracer uptake compared to those in sinus rhythm, and although 15% of the study cohort was in atrial fibrillation, the overall correlation with tracer uptake was strong. Aortic stenosis has been previously demonstrated to be associated with cardiac ATTR, which can also impact some echocardiographic parameters (30, 31). However, in the study group only one patient had mild aortic stenosis in the cardiac ATTR group and unlikely to make a significant impact on the outcomes.

^99m^Tc-DPD scintigraphy, initially utilized for bone scintigraphy, was found incidentally to demonstrate avidity for cardiac amyloid (16). A large multicentre trial demonstrated both high sensitivity and specificity for cardiac ATTR detection (32). The mechanism of ^99m^Tc-DPD binding to ATTR fibrils is yet to be identified (33). Although, some patients with AL amyloidosis demonstrate ^99m^Tc-DPD tracer uptake, the absence of monoclonal protein in serum and urine affords diagnostic sensitivity >99% and specificity 100% (32).

^99m^Tc-DPD scintigraphy image acquisitions vary, with methods using either thorax targeted or whole body planar images; more recently, SPECT-CT imaging has been employed (34). ^99m^Tc-DPD image assessment has been performed by qualitative visual grading using the Perugini method or semi-quantitative assessment with heart-to-body region ratios (15, 34). Incorporating ^99m^Tc-DPD SPECT-CT imaging has allowed exploration of quantitative methods to evaluate cardiac amyloid burden (16, 17). The Perugini method was evaluated previously as a prognostic tool, however did not demonstrate any survival difference between different grades (15). NT-proBNP is a significant predictor of mortality in ATTR patients (15). The correlation between ^99m^Tc-DPD tracer uptake and NT-proBNP levels suggest that quantitative ^99m^Tc-DPD scintigraphy could potentially provide prognostic information; however, this requires validation.

Echocardiography plays an important role in screening and longitudinal follow-up of cardiac ATTR patients (10, 35). Characteristic features of cardiac ATTR include biventricular increase in wall thickness and increased myocardial echogenicity (referred as a “speckled” appearance) (1, 7, 36). Diastolic dysfunction is present; however, LVEF is usually preserved until advanced disease (1). Cardiac ATTR strain analysis demonstrates subclinical systolic impairment despite preserved LVEF, with reduced basal and relatively preserved apical segmental longitudinal strain, producing the characteristic “apical sparing” strain pattern (1, 36). Increased LV wall thickness results in worsening GLS and eventually LVEF (7, 36). Previous studies have shown LVEF demonstrates lower sensitivity than LV GLS for identification of early or subclinical LV dysfunction, and this finding has been recapitulated in the present analysis where LVEF was preserved, with no correlation with tracer uptake (37, 38).

Only few studies evaluating echocardiographic changes in ATTR patients with ^99m^Tc-DPD tracer uptake employed a combination of qualitative visual grading, semi-quantitative analysis and planar imaging to quantify ^99m^Tc-DPD tracer uptake (39–41). In congruence with our findings, these earlier studies demonstrated ^99m^Tc-DPD tracer uptake correlates with LV wall thickness, mitral annular systolic velocity, tricuspid annular plane systolic excursion, E/e' and LV GLS (39–41). To our knowledge, our study is the first to quantitate ^99m^Tc-DPD tracer uptake in ventricular chambers and correlate this with biventricular structural and functional echocardiographic parameters. Additionally, prior studies focused primarily on LV parameters, with limited RV assessment, and only one previous study evaluated LV GLS (39–41).

Given the good correlation, we demonstrate the potential utility of TTE for longitudinal patient follow-up alongside quantitative ^99m^Tc-DPD scintigraphy for monitoring both disease progression and response to emerging therapeutic agents. Echocardiography could be particularly useful in regional centers, where availability of quantitative ^99m^Tc-DPD scintigraphy is limited.

### Study Limitations

Our sample size was limited to 40 patients with echocardiographic and ^99m^Tc-DPD scintigraphy data available; however, these are data from a single site and are comparable to other ATTR patient studies (39–41). The number of asymptomatic *TTR* variant carriers was small, but previous studies have also demonstrated no tracer uptake or evidence of echocardiographic abnormalities in such individuals (25). Larger studies with more patients are required to establish cutoff values for ^99m^Tc-DPD tracer uptake corresponding to traditional echocardiographic parameters. Further prospective studies with contemporaneous echocardiograms and ^99m^Tc-DPD scintigraphy would be required for validation. Our cohort included 16 patients with a history of mild hypertension with 14 in the cardiac involvement group, which may have contributed to some of the alterations in GLS. In our patients used to assess our reproducibility, seven of the 20 patients randomly selected had a history of mild hypertension, but still demonstrated an overall low variability. To determine the independent effects of comorbidities such as hypertension and diabetes mellitus, future studies with larger patient groups would be required for adequate subgroup analysis. Longitudinal studies are required to confirm whether abnormalities in these imaging parameters correlate with disease progression demonstrate response to specific treatment modalities and afford prognostic value.

## Conclusion

We have demonstrated an association between echocardiographic structural and functional biventricular parameters with quantitative ^99m^Tc-DPD scintigraphy uptake. Additionally, we demonstrated that absence of ^99m^Tc-DPD tracer uptake is associated with normal structural and functional echocardiographic measurements, suggesting absence of cardiac ATTR deposition. Thus, in addition to their diagnostic utility for cardiac ATTR ^99m^Tc-DPD scintigraphy and echocardiography may both play significant roles in longitudinal follow-up of ATTR patients for monitoring disease progression and evaluating response to therapy.

## Data Availability Statement

The raw data supporting the conclusions of this article will be made available by the authors, without undue reservation.

## Ethics Statement

The studies involving human participants were reviewed and approved by WSLHD HREC. Written informed consent for participation was not required for this study in accordance with the national legislation and the institutional requirements.

## Author Contributions

All authors have contributed to the design, data collection, analysis, and manuscript preparation.

## Conflict of Interest

The authors declare that the research was conducted in the absence of any commercial or financial relationships that could be construed as a potential conflict of interest.

## Publisher's Note

All claims expressed in this article are solely those of the authors and do not necessarily represent those of their affiliated organizations, or those of the publisher, the editors and the reviewers. Any product that may be evaluated in this article, or claim that may be made by its manufacturer, is not guaranteed or endorsed by the publisher.
